# Heat Shock Protein 90 (Hsp90) Expression and Breast Cancer

**DOI:** 10.3390/ph5091008

**Published:** 2012-09-12

**Authors:** Flora Zagouri, Evangelos Bournakis, Konstantinos Koutsoukos, Christos A. Papadimitriou

**Affiliations:** Department of Clinical Therapeutics, Alexandra Hospital, School of Medicine, University of Athens, 80 Vas. Sofias Ave, 11528 Athens, Greece; Email: vagimith@yahoo.com (E.B.); kkoutsoukos@yahoo.gr (K.K.); chr_papadim@yahoo.gr (C.A.P.)

**Keywords:** hsp90, breast cancer, 17-allylamino-17-demethoxygeldanamycin

## Abstract

Hsp90 is an abundant protein in mammalian cells. It forms several discrete complexes, each containing distinct groups of co-chaperones that assist protein folding and refolding during stress, protein transport and degradation. It interacts with a variety of proteins that play key roles in breast neoplasia including estrogen receptors, tumor suppressor p53 protein, angiogenesis transcription factor HIF-1alpha, antiapoptotic kinase Akt, Raf-1 MAP kinase and a variety of receptor tyrosine kinases of the erbB family. Elevated Hsp90 expression has been documented in breast ductal carcinomas contributing to the proliferative activity of breast cancer cells; whilst a significantly decreased Hsp90 expression has been shown in infiltrative lobular carcinomas and lobular neoplasia. Hsp90 overexpression has been proposed as a component of a mechanism through which breast cancer cells become resistant to various stress stimuli. Therefore, pharmacological inhibition of HSPs can provide therapeutic opportunities in the field of cancer treatment. 17-allylamino,17-demethoxygeldanamycin is the first Hsp90 inhibitor that has clinically been investigated in phase II trial, yielding promising results in patients with HER2-overexpressing metastatic breast cancer, whilst other Hsp90 inhibitors (retaspimycin HCL, NVP-AUY922, NVP-BEP800, CNF2024/BIIB021, SNX-5422, STA-9090, *etc*.) are currently under evaluation.

## 1. Introduction

Heat shock proteins (HSP) are members of the molecular chaperones, a group of proteins that play essential role in the folding of a large number of cellular proteins [1,2]. They were firstly discovered as mediators of resistance to hyperthermia [3]. Moreover, they participate directly in cell survival during hyperthermia by inhibiting programmed cell death and cell senescence [4,5,6,7]. HSPs appear to be utilized in carcinogenesis in order for cells to escape the pathways of tumour suppression, to promote progression in more advanced stage, to become treatment-resistant, and to facilitate metastasis [8].

The transcription of HSP genes is regulated by transcription factor HSF1; the latter senses cellular exposure to stress and turns on rapid induction of HSPs [9,10]. Moreover, HSF1 plays a putative role in breast carcinoma progression by inducing HSPs [11]. Additionally, HSF1 includes activation of metastasis by the silencing of anti-metastatic processes, activates pro-malignant signalling cascades and regulates the mitotic spindle checkpoint [12,13,14]. Hsp27 and Hsp70 appear to foster carcinogenesis by inhibiting apoptosis and senescence [8]. Mitochondria possess a subclass of HSPs including the Hsp70 homolog mortalin and TRAP-1 as well as Hsp60; all of them playing a key role in cancer progression [15,16].

Hsp90 is an abundant protein in mammalian cells [17]. It forms several discrete complexes, each containing distinct groups of co-chaperones that assist protein folding and refolding during stress, protein transport and degradation [18]. Hsp90 interacts with a variety of proteins that play key roles in breast neoplasia; including estrogen receptors (ER), tumor suppressor p53 protein, angiogenesis transcription factor HIF-1alpha, antiapoptotic kinase Akt, Raf-1 MAP kinase and a variety of receptor tyrosine kinases, such as HER2 (reviewed in [19]). This review article summarises the more significant points supporting the role of Hsp90 in breast carcinoma.

## 2. Hsp90 Biology

Hsp90 is highly conserved and expressed in a variety of different organisms from bacteria to mammals—including the prokaryotic analogue HtpG (high temperature protein G) with 40% sequence identity and 55% similarity to the human protein [20] Yeast Hsp90 is 60% identical to human Hsp90α.

In mammalian cells, there are two or more genes encoding cytosolic Hsp90 homologues [20], with the human Hsp90α showing 85% sequence identity to Hsp90β [21]. The α- and the β-forms are thought to be the result of a gene duplication event that occurred millions of years ago [20]. A membrane-associated variant of cytosolic Hsp90, lacking an ATP-binding site, has recently been identified and was named Hsp90N [22]. This HSP90α-Δ-*N* transcript is a chimera, with the first 105 bp of the coding sequence derived from the CD47 gene on chromosome 3q13.2, and the remaining coding sequence derived from HSP90AA1 [21]. However, gene-encoding Hsp90N was later proven to be non-existent in human genome. It is possibly a cloning artifact or a product of chromosomal rearrangement occurring in a single cell line [23].

Hsp90 consists of four structural domains [24,25,26]:

(1). A highly conserved *N*-terminal domain (NTD) of ~25 kDa
(2). A “charged linker” region, that connects the *N*-terminus with the middle domain
(3). A middle domain (MD) of ~40 kDa
(4). A *C*-terminal domain (CTD) of ~12 kDa.

The NH_2_-terminal domain consists of a highly twisted eight-stranded β sheet exposed to solvent on one side, whereas the other side is covered by helices that pack to form a pocket, which is the ATP-binding site [27]. Crystal structures are available for the *N*-terminal domain of yeast and human Hsp90 [27,28,29], for complexes of the *N*-terminus with inhibitors and nucleotides [27,28] and for the middle domain of yeast Hsp90 [30]. Recently structures for full length Hsp90 from *E. coli* (2IOP, 2IOQ) [31] yeast (2CG9, 2CGE) [32] and the dog endoplasmic reticulum (2O1U, 2O1V) [33] were elucidated [34]. Hsp90 forms homodimers where the contact sites are localized within the *C*-terminus in the open conformation of the dimer. The *N*-termini also come in contact in the closed conformation of the dimer [30].

HSP90 functions as a part of a multi-chaperone complex, involving the dynamic association with various accessory co-chaperones and client proteins [35]. In an ATP-bound state, HSP90 adopts a closed conformation and becomes a mature complex that is essential for it to perform its function of client protein folding and stabilization [35]. The chaperone cycle involves a complex series of loading and unloading events, which require a host of co-chaperones, such as HSP70, HSP40, HOP, AHA1, and p23 [36]. As already mentioned, HSP90 plays a putative role to the stability and function of a host of proteins such as BCR-ABL, HER2, epidermal growth factor receptor (EGFR), CRAF, BRAF, AKT, MET, VEGFR, FLT3, androgen and estrogen receptors, hypoxia-inducible factor (HIF)-1a, and telomerase; these protein play key roles in breast neoplasia such as growth factor independence, resistance to antigrowth signals, unlimited replicative potential, tissue invasion and metastasis, avoidance of apoptosis, and sustained angiogenesis [35,36]. Therefore, it is obvious why HSP90 should be considered an important molecular target for breast cancer.

All the HSP90 inhibitors in clinical development to date are focused on the ATP binding site of the *N*-domain [37]. Recent efforts have been directed to towards alternative approaches such as inhibition of the *C*-terminus [38], or inhibition of co-chaperones such as CDC37 [39], AHA 1 [40], HSP27 [41], HSP70 and HSF-1 [42].

In this point it should be underlined that inhibiting HSP90, multiple signal transduction pathways are combinatorial inhibited. HSP90 inhibitors are thought to be cytostatic agents and one way to increase their efficacy and to realize their full therapeutic potential would to use combinatorial approaches. For example because HSP90 inhibitors can lead to simultaneous disruption of many signal transduction pathways and therefore deliver a combinatorial effect through a single molecular target (HSP90); they can be combined with other biologic/targeted agents and thus prevent or reverse the resistance of these targeted agents [43]. Similarly, preclinical models have suggested additive/synergistic effects when HSP90 inhibitors are combined with conventional chemotherapy or radiotherapy [37,43].

## 3. Hsp90 Expression in Breast Carcinogenesis

Elevated Hsp90 expression has been documented in breast ductal carcinomas [44,45,46,47,48], whilst a significantly decreased Hsp90 expression has been shown in infiltrative lobular carcinomas and lobular neoplasia [49,50].

The persistent downregulation of Hsp90 expression throughout the whole lobular series (at the precursor and invasive components) may be contrary to what might have been expected; it is known that Hsp90 overexpression is a feature of invasive ductal carcinomas [45,46,47,51,52]. It is thus tempting to speculate that the whole lobular series may display a discrete, less intense profile of Hsp90 expression, which differentiates itself from the marked upregulation in ductal carcinomas. The underlying mechanisms of this discrepancy remain elusive.

According to our published results [48], ductal hyperplasia without atypia, atypical ductal hyperplasia (ADH) and ductal carcinoma in situ (DCIS) do not exhibit marked Hsp90 upregulation, while IDC presents with high Hsp90 expression. This finding may imply that the precursor context may not entail the cellular stress present in invasive cancer [50]. Consequently, as an integral part of the stress response, Hsp90 per se appears not to have been triggered early [48]. Within IDC, a higher grade, larger tumor size, higher ER expression and HER2 positivity correlate with higher Hsp90 expression [48]. More specifically, tumors of larger size also presented with more pronounced Hsp90 upregulation [47,48] which seems to reflect the stress-related events in rapidly proliferating hypoxic (due to their size) tumors. Significantly decreased Hsp90 expression has been observed in triple-negative tumours [48,53]. This may seem fairly rational as Hsp90 is associated with ER and HER2 expression. However, this observation highlights a paradox; on the one hand elevated Hsp90 has been suggested as a poor prognostic factor [53], whilst on the other hand triple-negative tumors, exhibiting decreased Hsp90 expression, are associated with poor prognosis (reviewed in [54]).

The discrepancy between HSP90 expression in IDC and ILC is rather very interesting. A high HSP90 expression in primary breast cancer has been described as a poor prognostic marker in breast cancer [47]. HSP90 expression was variable in patient tumors compared to cancer cell lines. Whether this expression is also variable between primary and metastatic tumors is unknown at this time. It is important to note that HSP90 inhibitors have been used in breast cancer only in the metastatic and refractory settings. Additionally, studies have not described HSP90 expression as a marker that can predict response to HSP90 inhibitor therapy. At the Annual ASCO meeting in June 2012 [55], a small recent retrospective study evaluated pre-treatment tumor specimens for biomarkers such as HSP90 and HSP70 expression and other client proteins (using immunohistochemical staining) that can predict response to HSP90 inhibitors. The sample size was too small to make any definitive conclusions; however, the most important predictive biomarker was HER2. HSP90 expression did not correlate strongly with response [55]. Prospective studies are ultimately needed to further elucidate the role of HSP90 expression and other predictive biomarkers of response to HSP90 inhibitor therapy.

Contrary to the pathological findings published by our team [48,49,50], as well as by other research groups [45,46,47,51], according to which Hsp90 exhibits significant variability along both series, such a finding was not replicated at the level of Hsp90 serum concentrations [56].

## 4. Hsp90 Inhibitors and Breast Cancer

Breast cancer is an indication where HSP90 inhibitors should be explored for a variety of reasons (reviewed in [35]). Firstly, inhibition of HSP90 degrades HER2, a client protein, and HSP90 inhibitors have shown activity in HER2-driven xenograft models [57]. Moreover, modulation of estrogen and progesterone receptor signaling has been a long-standing approach to treating breast cancer and both estrogen and progesterone receptors are clients of HSP90 [58]. Additionally, resistance of breast cancer cells to chemotherapy is known to involve the phosphatidylinositol 3-kinase pathway [59], which is modulated by HSP90 by virtue of one of its key signaling proteins (AKT) being a client protein of HSP90. Furthermore, inhibition of HSP90 has also been known to modulate angiogenesis of breast cancer xenografts [60]. Finally, expression of HSP90 has been shown to correlate with adverse clinical outcomes, further validating HSP90 as a target in breast cancer [47]. Given the above observation, it would appear that pharmacological inhibition of Hsps can provide therapeutic opportunities in the field of cancer treatment [61]; Hsp90 inhibitors include the natural products geldanamycin and radicicol as well as semisynthetic derivatives 17-*N*-Allylamino-17-demethoxygeldanamycin (17AAG) [62].

Geldanamycin, bind directly to the ATP-binding pocket in the *N*-terminal domain of Hsp90, blocks the binding of nucleotides to Hsp90. Geldanamycin causes the degradation of the steroid receptor, blocking the chaperone cycle at the intermediate complex, and preventing the release of the receptor from the Hsp90-complex [63]. Radicicol-based inhibitors have not entered clinical development.

17-*N*-Allylamino-17-demethoxygeldanamycin (17AAG) is the semi-synthetic derivative of Geldanamycin, exhibiting a less toxic profile, but with same therapeutic potential as Geldanamycin. It is the first HSP90 inhibitor that has been tested in clinical trials. The aforementioned agent decreases the concentration of client proteins and deregulates the client proteins like hsc, keratin 8, keratin 18, akt, c-raf1 and caveolin-1, resulting in inhibition of signal transduction [64].

The development of isoform-selective drugs that are targeted to particular members of the HSP90 family (DMAG-N-OXIDE), the favourable safety/toxicity profile as well as the promising preliminary results makes anti-HSP90 agents a new promising area in the oncology field.

The phase I study by Modi *et al*. [65] was done in advanced solid tumors. However, the majority of the patients enrolled on that trial were HER2 positive breast cancer patients and all responses were noted in this subset [65]. This led to the phase II trial of tanespimycin combined with trastuzumab in this patient population.

These results are in accordance with the one published by the same study group in the same study population, in a phase II trial [66]. The overall response rate was 22%, the clinical benefit rate [complete response + partial response + stable disease] was 59%, the median progression-free survival was 6 months (95% CI: 4–9), and the median overall survival was 17 months (95% CI: 16–28) [66].

Currently 17-AAG and 17-DMAG are not under clinical development but there are many other Hsp90 inhibitors (retaspimycin HCL, NVP-AUY922, NVP-BEP800, CNF2024/BIIB021, SNX-5422, STA-9090 *etc*.) with better pharmacological and toxicological properties that are currently under investigation, holding great promise in breast cancer treatment [67,68,69,70,71,72,73]. More specifically, retaspimycin HCL (IPI-504) has shown promising results with a safe toxicity profile and antitumor activity in a phase I trial in patients with relapsed or relapsed and refractory multiple myeloma [74], but it has shown modest results in a phase II trial in patients with castration-resistant prostate cancer; it would be tempting to see its activity in breast cancer patients [75].

Novel synthetic Hsp90 inhibitors based on diverse chemical scaffolds have been developed; it seems that they generally have an improved pharmacologic profile when compared to 17-AAG, especially with regard to their availability through synthesis, evasion of multidrug resistance (MDR)-mediated efflux, metabolic stability, water solubility and ease of administration, and retained biological activity over a wider panel of tumors [70,71]. The first synthetic Hsp90 inhibitor to enter clinic is CNF2024/BIIB021 [70]. There are currently several ongoing Phase I trial studies of oral CNF2024/BIIB021 in advanced solid tumors. As far as breast cancer is concerned CNF2024/BIIB021 is currently under evaluation alone or in combination with trastuzumab [76]. A second synthetic Hsp90 inhibitor to enter clinic is VER-52296/NVP-AUY922 [73]. A Phase I–II trial of intravenously administered AUY922 is currently open, with the Phase I portion of the trial recruiting patients with several types of cancer, whilst the Phase II portion is limited to patients with either HER2 positive or ER positive locally advanced or metastatic breast cancer [76].

Another active small-molecule Hsp90 inhibitor based on the 6,7-dihydroindazol-4-one scaffold is SNX-5422 [77]. SNX-5422 has been evaluated in a phase I trial in patients with refractory solid malignancies and lymphomas. In this study, no objective responses were observed, but long-lasting stabilizations were obtained. Although no clinically significant drug-related ocular toxicity was seen in this study, the development of SNX-5422 has been discontinued because of ocular toxicity seen in animal models and in a separate phase I study. Finally, STA-9090, is claimed to have a chemical structure unrelated to the ansamycin family of Hsp90 inhibitors, such as 17-AAG. This agent is currently enrolling patients in two dose-ranging phase I clinical trials in solid tumors [76]. Therefore, it seems that further research exploring this therapeutic interaction and the activity of HSP90 inhibitors is clearly warranted.

## 5. Hsp90 Biology and HSP90 Inhibitors in the Different Subtypes of Breast Cancer

As is the case with many other targeted agents, patient selection seems to be the major limitation to the success of HSP90 inhibitors. In this section Hsp90 biology as well as the implications of HSP90 inhibitors in the different subtypes of breast cancer are discussed.

Concerning HER2 positive breast cancer, it is known that HSP90 is required for the stabilization of essential components of EGFR and HER2 signaling (HER2, AKT, c-SRC, RAF and HIF-1α). HER2 is among the most sensitive client proteins of HSP90 [78,79], and HSP90 inhibition mediates degradation of HER2, as well as PI3K and AKT in HER2-overexpressing cancer cells [80]. Consequently, HSP90 inhibitors plus trastuzumab have significant anticancer activity in patients with HER2-positive, metastatic breast cancer previously progressing on trastuzumab [66]. This is in line with the preclinical studies that demonstrated that HER2 is one of the most sensitive client proteins of HSP90 inhibition, given that HSP90 is a crucial facilitator of oncogene addiction [81]. Moreover, important to note is that the phase II study of tanespimycin and trastuzumab did not evaluate pre-treatment HSP90 expression in tumor tissues from breast cancer patients [66]. Although a number of agents are in development for HER2 positive and ER-positive breast cancers, HSP90 inhibitors also represent therapeutic opportunities in other molecular subtypes.

Triple negative breast cancer (TNBC) is defined by the lack of expression of ER, PR and HER2 receptors and has a higher rate of distant recurrence and a poorer prognosis than other breast cancer subtypes. Unfortunately, the lack of expression of a credentialed therapeutic target in this subtype of breast cancer limits the effective treatment options. In pre-clinical models, TNBCs have been sensitive to Hsp90 inhibitors [82,83]. Similarly to HER2 positive tumors, TNBCs were sensitive to Hsp90 inhibition through down-regulation of components of the Ras/Raf/MARK pathway in preclinical and *in vitro* studies [83]. Being a central integrator of multiple pathways, activation of HSP90 may maintain the malignant phenotype, facilitate metastasis, and promote treatment-resistance under the stress of cancer therapy in multiple breast cancer subtypes. It has been suggested that Hsp90 up-regulation may be a sign of poor disease prognosis [47] and a recent study has demonstrated that co-expression of HSP90 and PI3K or expression of HSP90 in combination with the loss of PTEN were associated with significantly worse recurrence-free survival in patients with breast cancer [84].

Finally, HSP90 inhibitors may have a central role in ER-positive breast cancer tumors. It is known that aromatase inhibitor-resistant breast cancers do not rely on hormone-mediated signaling, but growth factor signaling is important for their growth. It has been found that the ERα can be phosphorylated and activated in a ligand-independent manner. This activation is mainly due to the cross-talk between the ERα and growth factor signaling pathways, such as insulin-like growth factor-I receptor and HER2-mediated signaling pathways. These growth factor signaling proteins are important for resistance to aromatase inhibitors and are also HSP90 client proteins [85]. Interestingly, Wong *et al*. [84] provided data to support the notion that HSP90 inhibitors may be an effective therapy to treat aromatase inhibitor-resistant breast cancers and that improved efficacy can be achieved by combined use of a HSP90 inhibitor and an AKT inhibitor.

## 6. Conclusions

In conclusion, it could be said that Hsp90 plays a key role in breast carcinogenesis. It forms several discrete complexes, each containing distinct groups of co-chaperones that assist protein folding and refolding during stress, protein transport and degradation. It interacts with a variety of proteins ER, tumor suppressor p53 protein, angiogenesis transcription factor HIF-1alpha, antiapoptotic kinase Akt, Raf-1 MAP kinase and a variety of receptor tyrosine kinases of the erbB family. Hsp90 overexpression has been proposed as a component of a mechanism through which breast cancer cells become resistant to various stress stimuli. Given the above observation, it would appear that pharmacological inhibition of HSPs can provide therapeutic opportunities in the field of cancer treatment. HSP90 inhibitors have been hypothesized to be active preclinically in a wide variety of tumor types but clinically have shown objective tumor responses in HER2 positive breast cancer and most recently in ALK+ lung cancer. Sensitive client proteins such as HER2 are very important. Finally it should be noted that prospective studies are needed to evaluate the role of HSP90 expression as a prognostic and predictive marker of response to HSp90 inhibition from pre-treatment tumor samples.
